# Supplementary material for: Vapour Pressure Deficit (VPD) Drives the Balance of Hydraulic-Related Anatomical Traits in Lettuce Leaves
Source: Plants (Basel). 2022 Sep 11;11(18):2369. doi: 10.3390/plants11182369 (PMC9502365; doi:10.3390/plants11182369)
Supplement: Supplementary file 1 [file plants-11-02369-s001.zip › plants-1877627-supplementary.pdf]

Supplemental materials

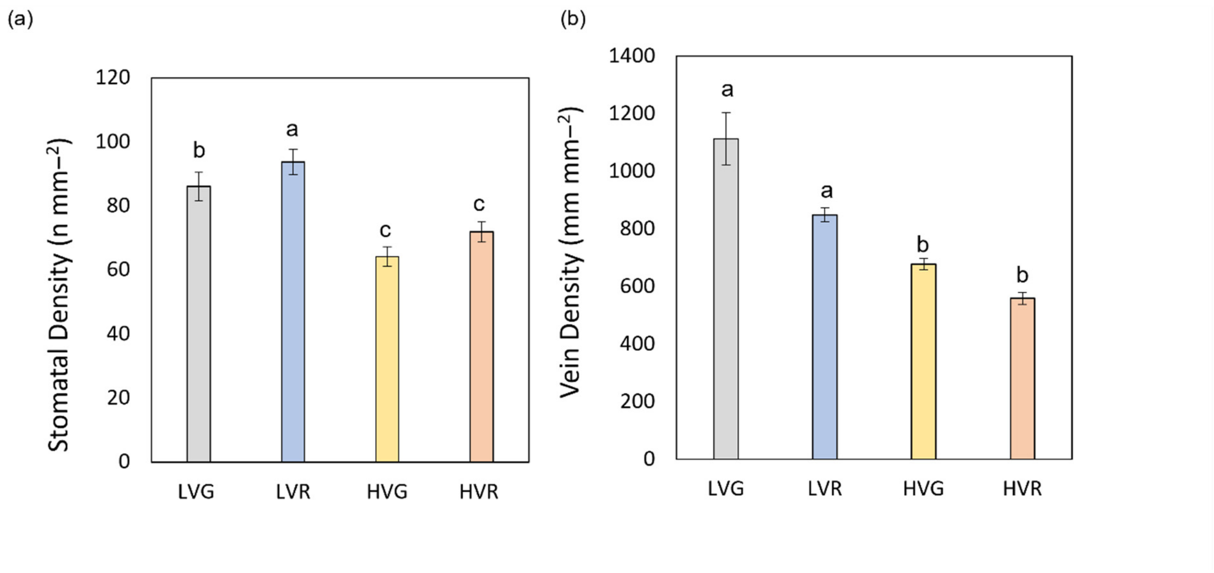

Figure S1. (a) stomatal density and (b) vein density of green and red Salanova lettuces grown under low and high VPD. Mean values ± standard errors are shown. Different letters correspond to statistically significant differences according to Tukey test ($p < 0.05$).

Table S1. Stomatal density per leaf size (SD x LS) and vein density per leaf size (VLA x LS) of green and red Salanova lettuces grown under low and high VPD. All data are reported as mean ± se. Different letters correspond to significant differences according to Tukey test ($p<0.05$).

	**SD X LS** (n)	**VLA x LS** (mm)
**VPD (V)**		
LV	126641 ± 607a	980 ± 10 a
HV	77963 ± 349b	618 ± 19 b
**Cultivar (C)**		
G	111253± 486 b	895 ± 29 a
R	113325 ± 471 a	703 ± 34 b
**Interaction**		
LVG	123269 ± 635 b	1113 ± 40 a
LVR	140130 ± 580 a	848 ± 24 b
HVG	73550 ± 338 c	677 ± 19 c
HVR	82376 ± 361 c	558 ± 20 c
**Significance**		
VPD	***	***
C	*	**
VPD x C	*	**

*NS, *, **, and ***, Not significant or significant at p < 0.05, 0.01, and 0.001, respectively.*

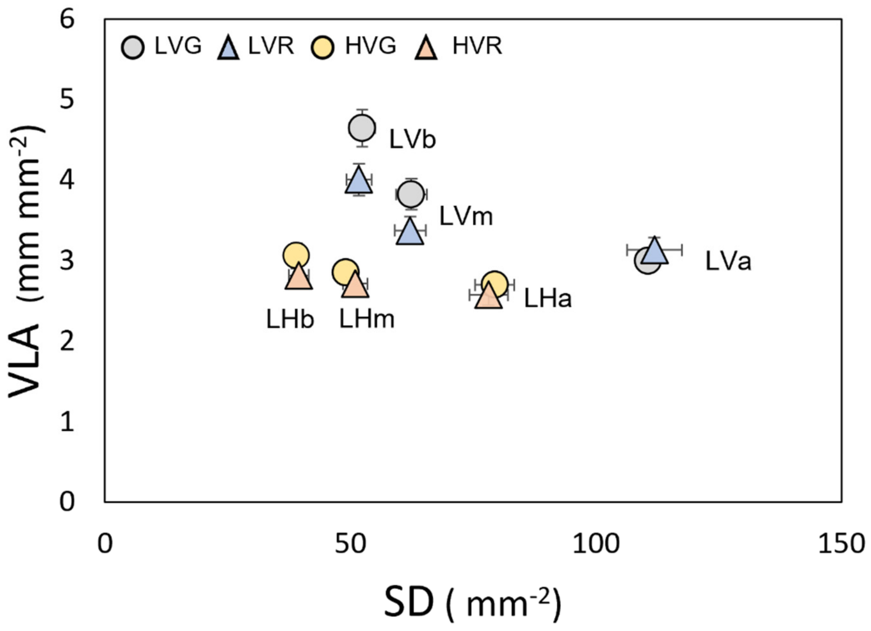

Figure S2. Mean values ± standard errors of vein and stomatal density relationships of grey (circles) and red (triangles) lettuce grown under low and high VPDs in the bottom (LVb and HVb), middle (LVm and HVm), and apex part (LVa and HVa) of the leaf.
